# Evidence for the association of chromatin and microRNA regulation in the human genome

**DOI:** 10.18632/oncotarget.20214

**Published:** 2017-08-12

**Authors:** Bang-Bao Tao, Xi-Qiang Liu, Wenhao Zhang, Shu Li, Dong Dong, Mang Xiao, Jun Zhong

**Affiliations:** ^1^ Department of Neurosurgery, Xinhua Hospital, Shanghai JiaoTong University School of Medicine, Shanghai 200092, China; ^2^ Department of Hepatobiliary-Pancreatic Surgery, Zhejiang Provincial People’s Hospital, Hangzhou 310014, China; ^3^ Department of Hematology, Xinhua Hospital, Shanghai JiaoTong University School of Medicine, Shanghai 200092, China; ^4^ Department of Pathophysiology, Wannan Medical College, Wuhu 241002, China; ^5^ Department of Otolaryngology Head and Neck Surgery, Sir Run Run Shaw Hospital, Zhejiang University, Hangzhou 310027, China; ^6^ Shanghai Key Laboratory of Regulatory Biology, Institute of Biomedical Sciences, School of Life Sciences, East China Normal University, Shanghai 200241, China

**Keywords:** microRNA, histone modification, DNA methylation, chromatin regulation, coordinated action

## Abstract

Both microRNAs (miRNAs) and chromatin regulation play important roles in cellular processes and they function at different regulatory levels of transcription. Although efforts have been devoted to the investigation of miRNA and chromatin regulation, there’s still no comprehensive work to illustrate their relationships due tothe lack of whole-genome wide datasets in different human cellular contexts. Based on the recently published large-scale epigenetic data, we examined the association between miRNA and epigenetic machinery. Our work confirmed a general relationship between miRNA biogenesis and chromatin features around pre-miRNA genomic regions. Obvious enrichments of DNA methylation and several histone modifications were observed within the pre-miRNA genomic region, which werecorrelated with miRNA expression levels. Furthermore, chromatin features at genepromoter regionsweretightly associated with miRNA regulation. Interestingly, we found that genes with their promoter regions located in the active chromatin state regions tend to have a higher probability to be targeted by miRNAs. This worksuggests that miRNAs and chromatin features are often highly coordinated, which provides a guide to deeply understand the complexity of gene regulation.

## INTRODUCTION

Mounting evidence have shown that miRNAs, an abundant class of small non-coding RNAs, are key post-transcriptional regulators of gene expressionin a wide variety of organisms ranging from plants to worms and mammals [1–4]. Recent studies have revealed various molecular mechanisms by which miRNAs down-regulate their target mRNAs [5, 6]. Around 30%- 80% of the human genes are predicted to be regulated by miRNAs [7–11]. Each miRNA can target multiple genes and, in turn, more than one miRNAs can bind a single mRNA target. It has been realized that the mechanism of miRNA regulation is quite complicated and needs to be scrutinized by the network-based systems biology approaches [12].

Recent studies have revealed that chromatin is one of the most complex molecular ensembles in the cell [13]. The eukaryotic DNA is tightly wrapped around histone octamers to form nucleosomes. Chromatin consists of arrays of nucleosomes with many dynamic features such as DNA methylation, post-translational histone modifications, as well as the binding ofchromatin-remodeling complexes and modification binding proteins, etc. [14–16]. It has been documented that chromatin features are involved in both activation and repression of transcription [17]. For example, H3K4me1 and H3K4me3 are tightly associated with transcriptional activation, while H3K27me3 and H3K9me3 are correlated with transcriptional repression [18–21]. The influence of chromatin on gene regulation is supported by the finding that histone post-translational modifications lead to the recruitment of protein complexes that regulate transcription [17, 20, 22].

Given the importance of miRNA and chromatin-regulation in the post-transcription regulation process, it is not surprising that miRNA and chromatin regulation are coordinated. Amongthe several regulatory mechanisms, one is based on epigenetic modifications. Recently, it has been proved that miRNA genes are subject to hyper-methylation and hypo-methylation in a tumor- and tissue- specific manner [23]. On the other hand, epigenetic features of specific genes are also correlated with miRNA regulation. For example, miR-148 has been shown to target *DNMT3B* gene [24], reflecting a regulatory feedback loop between epigenetic regulation and miRNAs. In this way, miRNA-epigenetic machinery forms an intricate network regulating gene expression.

Thanks to the wealth datasets from the ENCODE project [25], which opens the door for us to comprehensively explore the relationship between miRNAs and chromatin features in different human cellular contexts. In this study, we employed chromatin accessibility, DNA methylation and different types of histone modification data generated by the ENCODE project in six human cell lines to analyze their relationship to miRNAs. Our work revealed severalnew insights: (1) certainchromatin features around pre-miRNA regions weretightly associated with miRNA expression in different cell lines; (2) the promoters of miRNAs target genes were preferentially located in ‘open’ chromatin domains; (3)miRNA target gene promoters were negatively correlated with DNA hypomethylation; (4) active histone modification marks of gene promoter regions showed different patterns between miRNA targets and non-targets. These results provided a more comprehensive view ofthe relationship between miRNA and chromatin features. Abetter understanding of the relationshipbetweenmiRNA and chromatin regulation will help us to understand the complexity of transcriptional regulation.

## RESULTS

### Chromatin features can significantly influence miRNA transcription

Chromatin features have been thought to regulate the transcription of miRNA genes in a manner similar to that of protein-coding genes [26]. To comprehensively study the relationship between chromatin regulation and miRNA expression in multiple human cellular contexts, we characterized12 genome-wide chromatin tracks, including 10tracks of histone modification marks, one track of DNA methylationand one track of chromatin accessibility in six human cell lines (see Material and Methods). These data derived from extensive experimentswere performed by different ENCODE production groups, enabling integration across various types of chromatin features in different cellular contexts.

**Figure 1:**
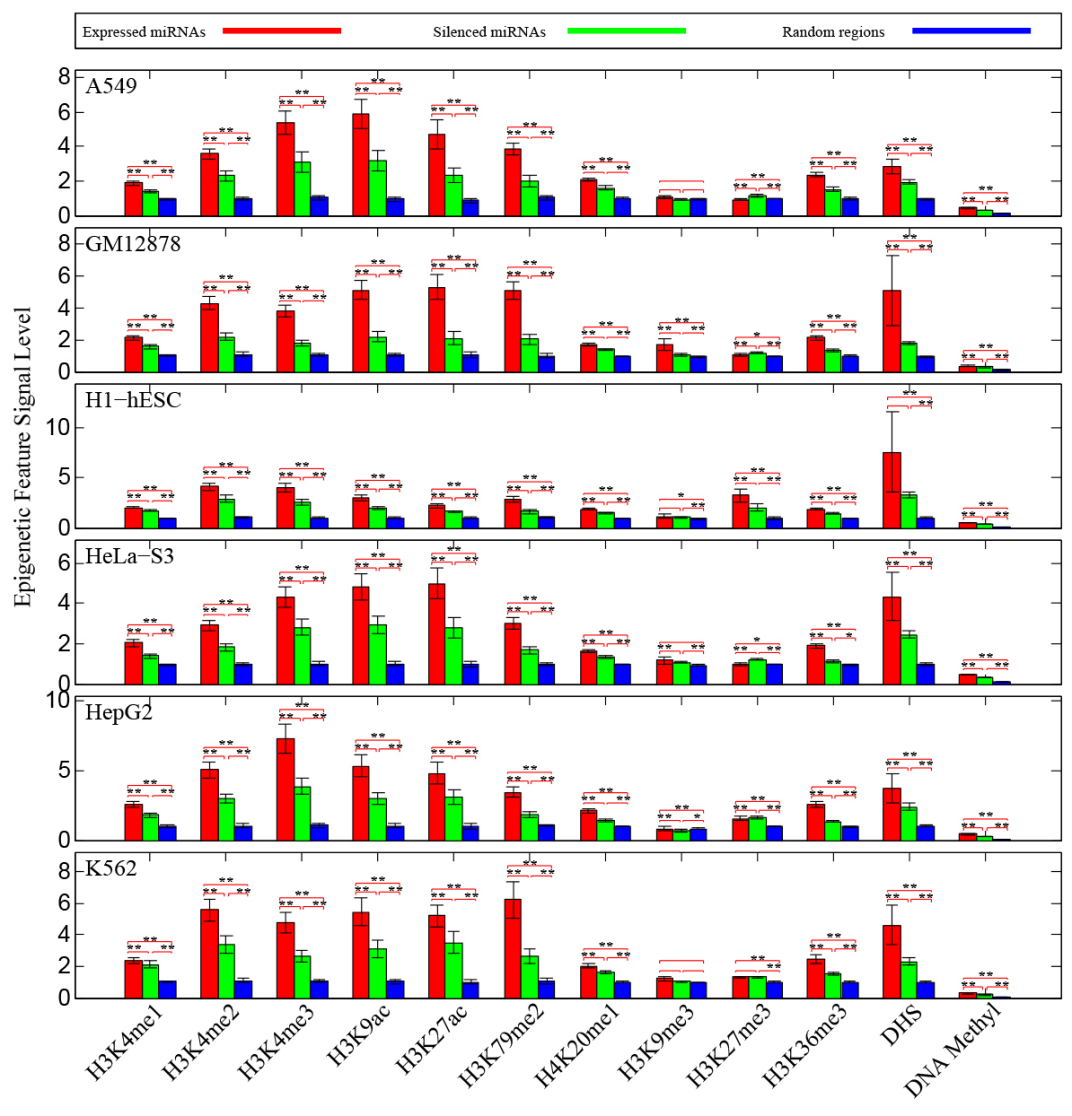
Enrichments of chromatin features around pre-miRNA sequences We compared the observed prevalence of chromatin features in pre-miRNA sequences against random sequences control. Statistical significances were assessed by *Wilcoxrank-sum* test, and error bars were estimated by using 95% confidence intervals. ‘*’ represents *P-value*< 0.05 and ‘**’ represents *P-value*< 0.01.

**Figure 2:**
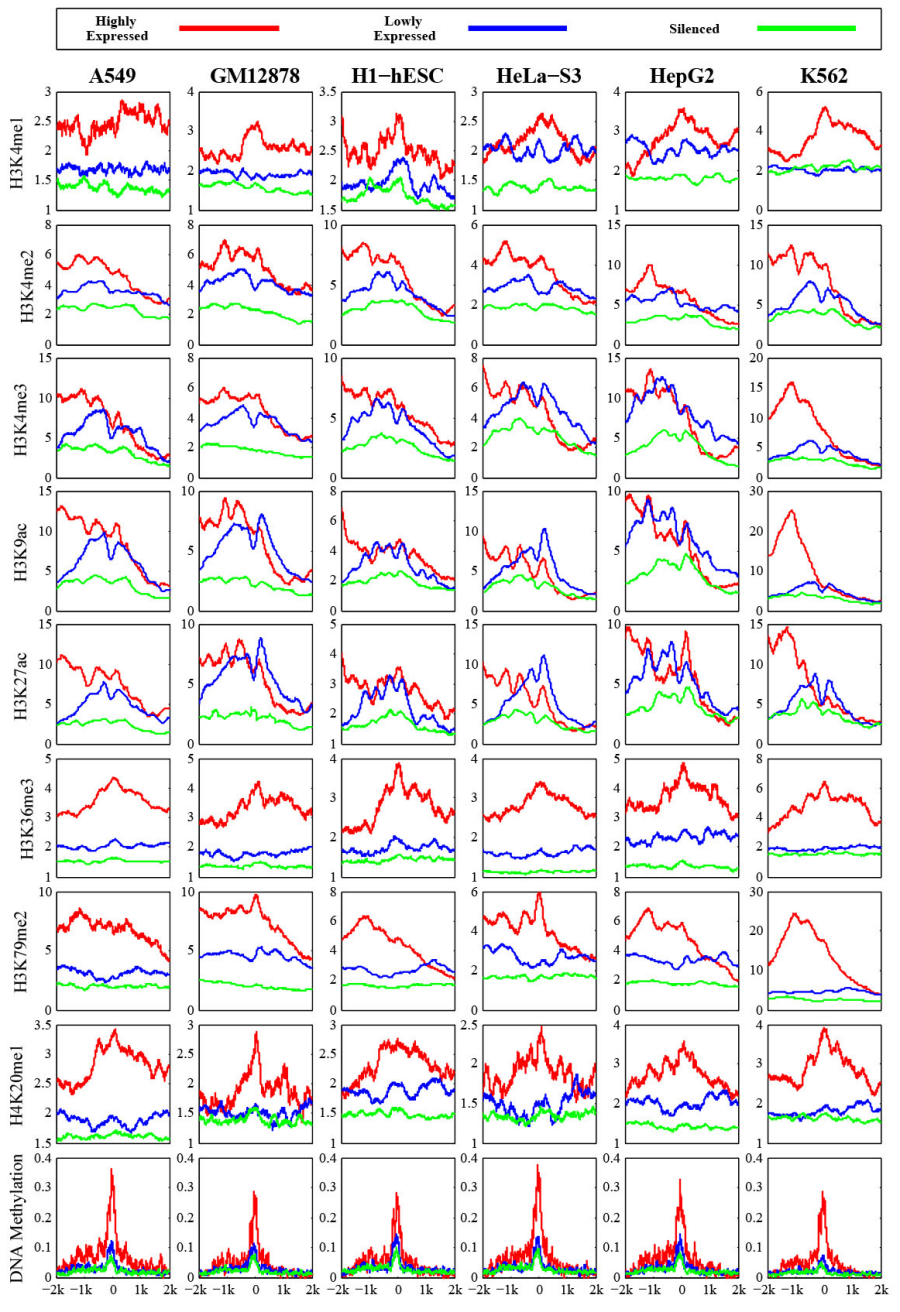
Profiles of chromatin features along pre-miRNA genomic sequences in human embryonic stem cell The y axis represents the average values of chromatin feature coverage, and the x axis represents the distance relative to the center of pre-miRNA.

At first, we measured chromatin features enrichment around human pre-miRNA genomic sequences. Average levels of histone modifications were calculated across the 4,000 bp window surrounding the center of each pre-miRNA sequence. We found obviously higher signals of different chromatin featuresin expressed miRNAs around the pre-miRNA sequencesthan expression-silenced miRNAsand random sequencesfrom intergenic regions (Figure 1), suggesting that chromatin features are highly enriched within microRNA precursor sequences. Next, we plotted the distributions of chromatin features around pre-miRNA sequences. Obvious enrichments of several chromatin features were observed in expressed miRNAs (Figure 2), particularly in highly expressed miRNAs in comparison to lowly expressed miRNAs and silenced miRNAs in human embryonic stem cells. The same conclusions were reached in all six human cell linecontexts. Notably, these activation associated histone marks significantly occupiedhighly expressed miRNAs.Furthermore, consistent with previous work [23], it was apparent that DNA methylations around pre-miRNA regions werecorrelated with miRNA expression levels (Figure 2). These results further validated the tight relationship between chromatin regulation and miRNA biogenesis.

**Figure 3:**
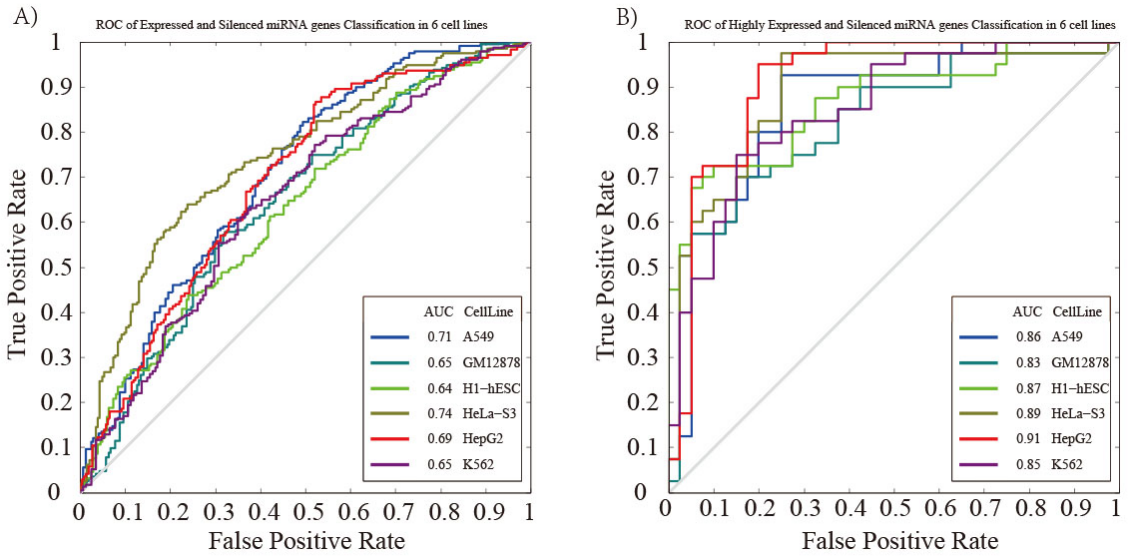
Prediction of miRNA expression using SVM classifier ROC curve is generated based on **(A)** the expressed and silenced miRNAs, and **(B)** highly expressed and silenced miRNAs. The gray line represents the ROC curve from randomly guessing.

To further investigate the correlation of chromatin features and miRNA expression, we combined these chromatin features around pre-miRNAs genomic sequences and used them as input features. Based on the expressed and silenced miRNA groups, we applied the SVM classifier model for predicting miRNA expression. The performance of the model was assessed by cross-validation (see Materials and methods). The result indicated that our model achieved a comparable information when predicting miRNA expression (measured by the area under the receiver operator characteristic curve, AUC) in different cell lines (Figure 3A, AUC_*H1-hESC*_=0.64, AUC_*GM12878*_=0.65, AUC_*HepG2*_=0.69, AUC_*K562*_=0.65, AUC_*HeLa-S3*_=0.74, AUC_*A549*_=0.71, respectively). When considering the highly expressed and silenced miRNA group, we obtained a higher level of classification accuracy with the AUC values ranging from 0.83 to 0.91 (Figure 3B, AUC_*H1-hESC*_=0.87, AUC_*GM12878*_=0.83, AUC_*HepG2*_=0.91, AUC_*K562*_=0.85, AUC_*HeLa-S3*_=0.89, AUC_*A549*_=0.86, respectively). Our results comprehensively indicated that it’s not a causal effect of chromatin features on miRNA expression, and chromatin features are involved in miRNA transcription.

### Correlation between miRNA regulation and chromatin features of gene promoter regions

Next, we examined the association betweenchromatin and miRNA regulation. Because the expressions of miRNAs are highly variable across different human cell lines, only expressed miRNAs in each cell line were considered. Previous works reported that gene expression was related to histone modification in their promoter regions [17, 27]. Therefore, the relationship between miRNA regulation and chromatin features (chromatin accessibility, DNA methylation and histone modification) of the gene promoter regions(defined as 4,000 bp window relative to the transcription start site) were investigated.

### miRNAs preferentially target genes with open chromatin domain in their promoters

**Figure 4:**
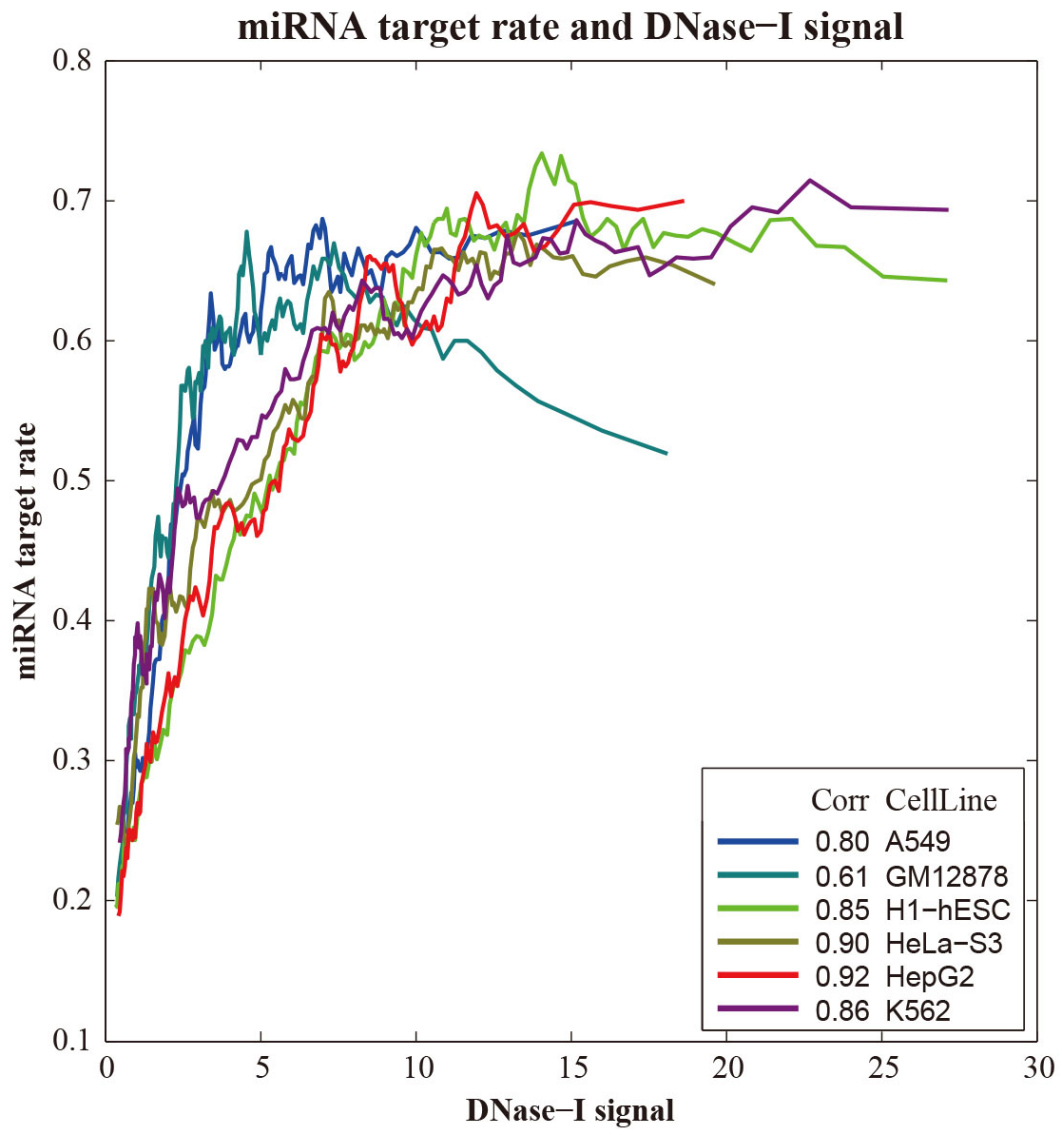
Correlation between DNase I hypersensitive sites and miRNAs in gene regulation in six human cell line

In order to investigate the relationship between miRNA regulation and chromatin accessibility, recent published DNase I hypersensitive sites (DHS) data generated by DNase-Seq method were compiledin these six human cell lines. Weexamined the average DNase signals within the promoter regions to the miRNA targets and non-targets, respectively. The result showed that DHS peaks were preferentially located in the promoter regions of miRNA targets than non-targets (Table 1).SinceDNase I sensitivity provides a quantitative marker of regions of open chromatin, we grouped all genes based on theDNase I hypersensitive signals in their promoter regions and calculating the miRNA target rate in each group. As shown in Figure 4, DNase I hypersensitive signals weresignificantly correlated with the miRNA target rate in all six human cell lines (R_*H1-hESC*_=0.68, R_*GM12878*_=0.69, R_*HepG2*_=0.73, R_*K562*_=0.63, R_*HeLa-S3*_=0.74, R_*A549*_=0.7, respectively). These results indicated that genes with their promoter regions exposed to the open chromatin state are more proneto be targeted by miRNAs.

**Table 1:** The average chromatin feature signals of promoter regions between miRNA targets and non-targets (targets/non-targets)

	A549	GM12878	H1-hESC	Hela-S3	HepG2	K562
**DHS**	0.13/0.10 (2.0E-43)	0.14/0.12 (6.1E-21)	0.35/0.25 (6.0E-112)	0.17/0.14 (1.2E-31)	0.23/0.19 (4.7E-42)	0.17/0.14 (2.9E-32)
**DNA methylation**	0.26/0.34 (1.1E-71)	0.21/0.28 (3.8E-71)	0.22/0.36 (2.7E-143)	0.35/0.40 (8.9E-19)	0.22/0.27 (3.7E-40)	0.19/0.21 (3.0E-06)
**H3K4me1**	2.28/1.92 (3.2E-49)	2.74/2.58 (8.8E-12)	3.26/2.62 (4.3E-86)	3.10/2.67 (1.0E-20)	3.62/3.41 (1.8E-09)	3.66/3.35 (5.2E-07)
**H3K4me2**	10.97/7.75 (2.0E-107)	10.36/8.33 (1.4E-47)	15.15/10.31 (2.2E-153)	8.57/6.61 (5.6E-41)	16.05/12.38 (2.7E-50)	17.78/14.25 (2.9E-26)
**H3K4me3**	21.50/14.58 (1.3E-96)	9.72/7.65 (2.4E-49)	14.46/10.06 (1.6E-108)	16.41/12.05 (1.2E-51)	26.61/19.20 (9.9E-63)	13.73/10.98 (7.5E-25)
**H3K9ac**	19.66/13.52 (6.3E-79)	12.74/9.90 (5.4E-34)	7.05/5.11 (1.9E-78)	16.51/12.03 (1.2E-40)	16.77/12.07 (1.1E-39)	14.23/10.99 (9.3E-20)
**H3K27ac**	11.47/7.73 (1.4E-62)	10.65/8.22 (2.0E-32)	3.79/2.90 (3.8E-45)	15.10/10.84 (8.3E-35)	14.32/10.25 (2.5E-28)	13.70/10.47 (1.2E-18)
**H3K79me2**	6.10/3.96 (2.0E-74)	9.39/6.85 (7.3E-39)	4.65/2.91 (3.2E-91)	5.30/3.81 (3.2E-43)	6.01/4.19 (1.4E-41)	10.85/7.76 (3.4E-25)
**H4K20me1**	1.86/1.50 (4.0E-69)	1.58/1.40 (2.7E-33)	1.91/1.49 (1.1E-133)	1.37/1.23 (1.0E-11)	1.93/1.40 (8.1E-91)	2.06/1.74 (6.5E-38)
**H3K9me3**	0.79/0.83 (0.83)	1.12/1.11 (0.66)	0.78/0.86 (4.2E-09)	0.86/0.89 (0.09)	0.46/0.48 (0.96)	1.06/1.09 (0.3)
**H3K27me3**	1.45/1.44 (086)	1.54/1.29 (8.0E-07)	5.94/3.68 (1.0E-19)	0.88/1.00 (1.4E-10)	3.08/3.54 (4.2E-26)	1.41/1.45 (0.075)
**H3K36me3**	1.40/1.29 (1.4E-16)	1.01/0.99 (0.34)	1.03/1.04 (0.78)	0.94/0.92 (0.49)	1.06/1.03 (6.7E-05)	1.52/1.46 (0.0063)

The values in the parenthesis are the *P-values* calculated by the *Wilcoxon signed-rank* test.

### miRNAs preferentially target genes with low DNA methylation level in their promoters

Next, we attempted to explore the relationship between promoter methylation and miRNA regulation by taking advantage of the recently published human methylome data in six human cell lines. In this study, we found that the promoter DNA methylation levels of miRNA targets weresignificantly lower than those of miRNA non-targets in six cell lines, indicating their functional complementation (Table 1). The ratio of the observed to the expected CpG content (CpG_o/e_) has been used as a proxy for the DNA methylation status in the human genome. Accordingly, genes were classified into hyper-methylated group and hypo-methylated group according to the extent of CpG_o/e_, so that hyper-methylated group hadlower-than-expected CpG_o/e_ and hypo-methylated group hadhigh CpG_o/e_. We found that DNA methylation levels in hyper-methylated promoters hadno significant differences between miRNA targets and non-target.

### miRNA targets are significantly correlated with active histone modification marks

**Figure 5:**
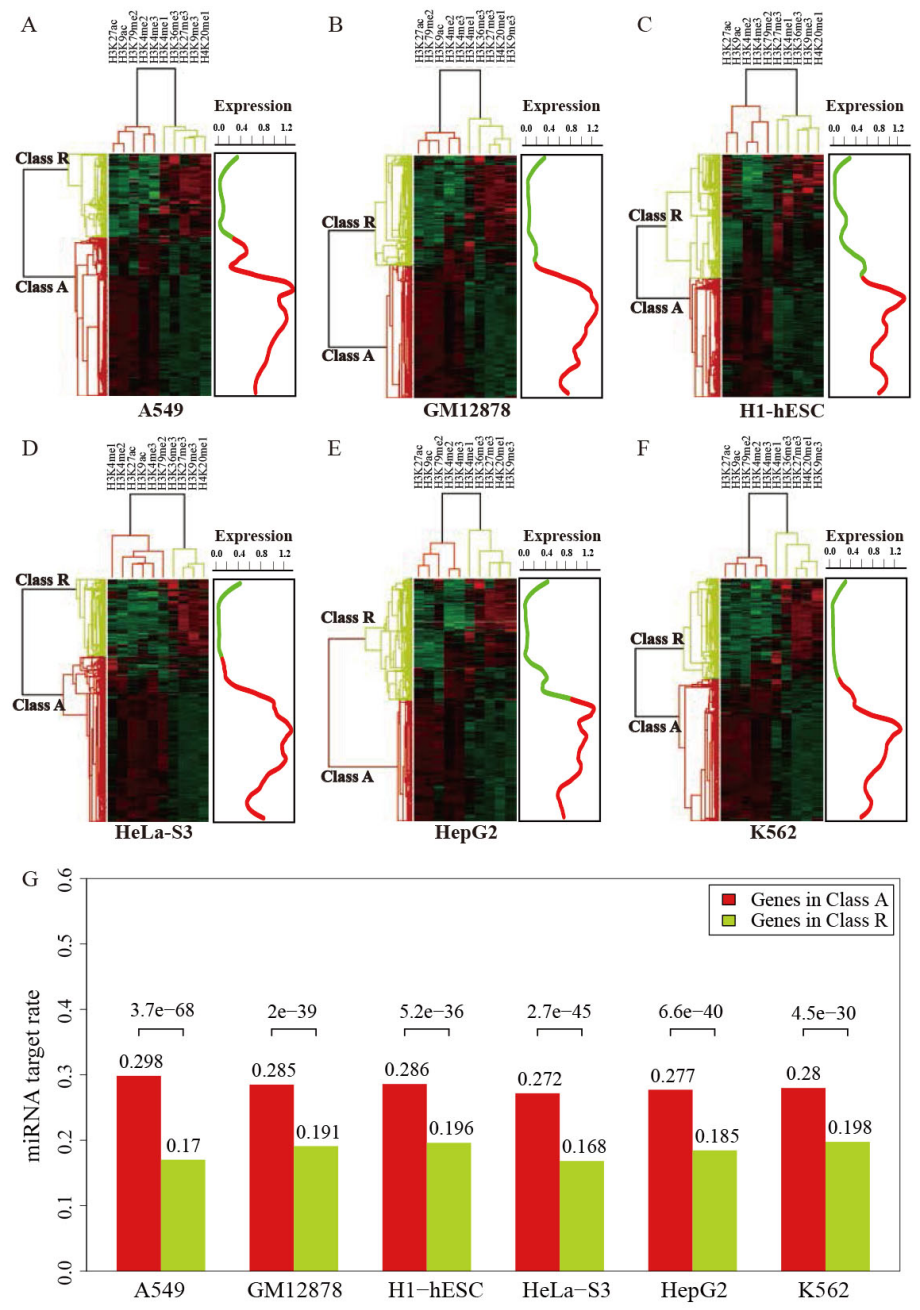
Two clusters of genes regulated by histone modifications in six human cell lines **(A-F)**, and the expression levels of the genes in cluster A and cluster R (right panel). Color density indicates the strength of histone modification signals in each cell lines. **(G)** miRNAs preferentially target genes in cluster A.

To better understand the relationship between histone modifications and miRNA regulation, we examined the enrichment profiles of 10 histone modification marks generated by ChIP-seq method around gene promoter regions between miRNA targets and non-targets.Except for H3K9me3, H3K27me3 and H3K36me3, We found that histone marks were significantly more enriched in the promoter regions of miRNA targets than miRNA non-targetsacross different cellular context (Table 1). H3K36me3 is associated with transcribed regions, while H3K9me3 and H3K27me3 are both considered marks for transcriptional repression. Since histone marks exhibit combinatorial patterns in the human genome [28], we used a hierarchical clustering method to analyze the activation and repression histone modification patterns of gene transcription. In each cell line, genes were segregated into active cluster (cluster A) and repressed cluster (cluster R), respectively. It was obvious that genes in cluster A correspondedto activating histone marks (such as H3K4me3 and H3K27ac) and hadhigher expression levels, whereas cluster R having more repressed marks (such as H3K9me3 and H3K27me3) tendedto be lowly expressed.Our result indicated that genes nested in cluster A are preferentially targeted by miRNA (Figure 5), which suggested the associations between miRNA regulation and histone modifications corresponding to gene activation.

## DISCUSSION

Transcription is a complicated dynamic process, involving a combination of various regulators. In this study, we undertook a comprehensive analysis of the relationship between miRNA and chromatin features across multiple human cell line, and we found that chromatin features wereassociated with both miRNA biogenesis and post-transcriptional regulation.Chromatin states and miRNAs are principle classes of gene regulators in transcription. The epigenetic landscape can determine the chromatin structural states that ultimately control the transcriptional outcome of the cell accommodate developmental or environmental requirements. Our work indicated an interconnection between miRNAs and chromatin machinery, which could provide a useful starting point to explore the molecular basis of morphological complexity.

Although the pathological and physiological importance of miRNAs has been appreciated, little is known regardingtheir regulation. Up to date, mounting evidence have indicated that a substantial number of miRNA genes are subjected to epigenetic alterations [29, 30]. An extensive analysis of miRNAs has shown that most of them are associated with CpG islands, suggesting that they are subjected to the regulation of DNA methylation [31]. Furthermore, several lines of evidence have proved that aberrant methylation status can be responsible for the deregulated expression of miRNAs in cancers [32]. Our results indicated that nearly all chromatin features are highly enriched in pre-miRNA regions, and some specific histone modifications and DNA methylations are associated with miRNA expression. Similar to protein-coding genes, we found that chromatin features are predictive of miRNA expression, suggesting some similarities between mRNA maturation and miRNA biogenesis. However, the performance of our miRNA expression prediction model is less accurate than protein-coding gene expression prediction, this can be partially attributed to the factthat miRNA biogenesis is also under the control of other regulatory mechanisms, such as transcription factor [33], etc.

The influences of miRNAs on target gene expression can be roughly classified into two different types: ‘tuning’ and ‘buffering’ [34]. In expression tuning, miRNAs relate to the expression level of their targets, whereas expression buffering relates to the reduced expression variance.The regulation of miRNA is a complicated process, and the association betweenmiRNAs and chromatin regulation leads to a more complicated scenario during transcription process. Recent works have documented that gene regulatory networks are always composed of some small sets of recurring interaction patterns called ‘motifs’ [35, 36]. In cases studies so far, these network motifs are likely to preserve their phenotypes, wired into the regulatory networks of the cell.In this work, we provided evidence of associations between miRNAs and chromatin regulation. Both chromatin features and miRNAs can exert a widespread impact on gene expression, and the miRNAs expression and their post-transcriptional regulation are influenced by chromatin regulation. They present a prevalence of integrated transcriptional regulatory circuit. Posttranscriptional control of expression variation is carried out by miRNAs, such that the miRNA and target genes are wired into an incoherent feed forward loop. Within such incoherent feed forward loop architecture, miRNAs can buffer expression variation of their target genes against the fluctuation of chromatin features.The incoherent feed forward loop is characterized as one of the most common network motifs in transcription networks, which is largely dominated in both bacteria [37] and fungi [38].According to this setup, we might expect that chromatin features and miRNAs mediated mechanism can maintain homeostasis and increase network robustness.

Taken together, our work provided a comprehensive investigation on the miRNA-epigenetic relationship. The results suggest that these two principles of gene regulations are not entirely separable, and a complicated mechanism might tie it all together. We speculated that the emerging pictures of transcription regulation are much more complicated than previously thought.This study comprehensively provided the first attempt to understand the complexity of gene regulation control.

## MATERIALS AND METHODS

### Epigenetic data sources

We compiled 12 epigenetic features in six human cell lines, consisting of embryonic stem cells (H1-hESC), B-lymphoblastoid cells (GM12878), hepatocellular carcinoma cells (HepG2), erythrocyticleukaemia cells (K562), epithelial carcinoma cells (HeLa-S3) and alveolar basal epithelial cells (A549). All data used in this work were downloaded from the University of California, Santa Cruz (UCSC) hg19 genome browser (http://genome.ucsc.edu/encode/). Histone modifications within the histone tails were compiled, and these data were identified by ChIP-seq method generated by the ENCODE project. This dataset were generated using the same platform, containing 10 histone modifications in each cell line (H3K4me1, H3K4me2, H3K4me3, H3K27me3, H3K36me3, H3K9me3, H3K79me2, H3K20ac, H3K27me1 and H3K9ac).DNase I hypersensitivity is an alternative measurement of chromatin accessibility, and DNase-Seq provided a powerful technique for identifying genome-wide DNase I hypersensitive sites [39]. In order to determine whether mRNAs targeted by miRNA are preferentially located in open chromatin domains, we compiled DNase I hypersensitive site data generated by DNase-Seq method from different cell lines. DNA methylation data were also downloaded from UCSC genome browser, and these data were all generated using 450K methylation BeadChip.

### Annotation of pre-miRNAs, miRNAs and their target genes

We downloaded the annotation of 1,594 human small hairpin precursor (pre-miRNA) from miRBase (http://www.mirbase.org/) [40], and explored the distribution of these chromatin features across4,000 bp windows surrounding the centers of pre-miRNAs genomic sequences. Mature miRNAs (2,233) were also retrieved from miRBase, and their expression levels were quantified using the next-generation sequencing method generated by the ENCODE project.

In this study, we used three current *in silico* miRNA target prediction methods to determine miRNA target genes, including TargetScan [41], PITA [42] and Pictar [43].To minimize the false positive of miRNA target prediction, a high-quality miRNA target data set was generated by intersecting data generated by at least two different *in silico* miRNA target prediction methods. Those without being detected by any method were defined as miRNA non-targets. In this work, miRNA target rate was defined as the ratio of the number of genes that were miRNA targets to the total number of genes in human.

### Support vector machine model for miRNA expression prediction

Based on the miRNA expression data, miRNAs could be classified into expressed (sequencing reads can be detected) and silenced groups. Furthermore, expressed miRNAs were classified into highly and lowly expressed groups using *K*-means clustering method. Chromatin features across 4,000 bp windows surrounding the centers of pre-miRNA sequences were integrated. Support vector machine (SVM), implemented by LibSVM package [44], was then introduced for classification. We evaluated the performance of the models using two-fold cross-validation. Briefly, we randomly divided the data into two subsetswith equal sizes, one training set and one testing set, respectively. The model was trained using the training set and applied to the testing set to predict expression. The prediction power of the SVM model was estimated based on the testing set. The model generates a probability indicating how likely a miRNA is to be expressed. By setting different threshold values, we can depict the sensitivity (true positive rate) and the specificity (true negative rate) of the prediction. Receiver operator characteristic (ROC) curve was used to show the classification accuracy of our SVM model.
